# Ca^2+^/calmodulin-dependent protein kinase II regulates the inflammatory hDPSCs dentino-differentiation via BDNF/TrkB receptor signaling

**DOI:** 10.3389/fcell.2025.1558736

**Published:** 2025-03-26

**Authors:** Ji Hyun Kim, Muhammad Irfan, Sreelekshmi Sreekumar, Kerwin Chong, Jin Hong, Satish Alapati, Seung Chung

**Affiliations:** ^1^ Department of Oral Biology, College of Dentistry, University of Illinois Chicago, Chicago, IL, United States; ^2^ Department of Endodontics, College of Dentistry, University of Illinois Chicago, Chicago, IL, United States

**Keywords:** CaMKII, TrkB, inflammation, TNFα, hDPSCs, odontoblastic differentiation

## Abstract

CaMKII is a serine/threonine-specific protein kinase that plays a crucial role in normal and pathological conditions. However, limited information is available regarding the roles of CaMKII in dentinogenesis, particularly in an inflammatory context. Previously, we demonstrated the pivotal role of TrkB in inflammation-induced differentiation of hDPSCs into odontoblast-like cells. Here, we investigate the interaction between CaMKII and TrkB during hDPSCs odontogenic differentiation. hDPSCs were cultured and subjected to CaMKII knockdown using siRNA, followed by treatment with dentinogenic media. TNFα-stimulated cells were treated with CaMKII- inhibitor, -protein, or TrkB antagonist, CTX-B. Immunocytochemistry and ARS were used to visualize targeted proteins and calcium deposits. Real-time PCR detected expression levels of odontogenic and mineralization markers such as DSPP and DMP-1. Our data indicate that CaMKII inhibition enhances TrkB protein levels and promotes TNFα-induced transcriptional activation of genes associated with odontogenic differentiation. CaMKII knockdown via siRNA and pharmacological inhibition elevated DSPP and DMP-1 protein levels, whereas CaMKII overexpression suppressed their expression. Notably, treatment with TNF-α and a CaMKII inhibitor upregulated DSPP and DMP-1 expression, while co-treatment with CTX-B abolished this effect. Similarly, mRNA expression of DSPP and DMP-1 was reduced at day 10. Mineralization activity exhibited a similar pattern to the expression of these markers. Our findings unveil a novel mechanism underlying the role of CaMKII via TrkB in dentinogenesis, which is vital for the success of hDPSCs engineering strategies.

## 1 Introduction

The dental pulp is a soft tissue localized within the tooth structure surrounded by hard tissue dentin. Pulp tissue predominates composed of connective tissue, blood vessels, and neural fibers derived from mesenchymal, ectodermal (ectomesenchyme), and neural crest origin (Lan et al., 2019). Its primary functions include dentin formation, providing nutrition to the dentin, innervation, and assisting in tooth defense (Slavkin, 1978; Ghannam et al., 2023). Upon progression of microbial infection, inflammation within the pulp/dentin triggers differentiation of dental pulp stem cells to the injured site and forms tertiary dentin to promote regeneration (Irfan et al., 2022a). Human dental pulp stem cells (hDPSCs) are self-renewing mesenchymal stem cells located within the pulp’s vasculature and can be harvested from the tissue. Like bone marrow mesenchymal stem cells, hDPSCs can differentiate into various cell types, including dentin-forming cells, osteoblasts, chondrocytes, adipocytes, endothelial cells, and neurons *in vitro* under specific conditions (Ferro et al., 2014). Their significance extends to the realms of regenerative medicine and regenerative dentistry.

In dentin regeneration, inflammation has been shown to enhance the cellular proliferation of hDPSCs. Studies have revealed a higher number of hDPSCs in pulpal tissues of patients with reversible pulpitis and deep caries lesions (Abd-Elmeguid et al., 2013). *In vitro*, the inflammatory cytokine Tumor Necrosis Factor Alpha (TNF-α) has been found to stimulate the cellular proliferation of hDPSCs; however, excessive activation of TNF-α can lead to chronic inflammation and cell death (El-Sayed et al., 2019). Thus, inflammation is a critical factor to consider in dentin-pulpal complex regeneration. We previously reported that brain-derived neurotrophic factor (BDNF) and its receptor, tropomyosin-related kinase B (TrkB) activation, enhances TNFα-stimulated odontoblastic differentiation of hDPSCs, suggesting a significant role in promoting tertiary dentin formation. While other bacterial components such as Lipoteichoic Acid (LTA) and Lipopolysaccharides (LPS) have also been shown to stimulate the growth of hDPSCs, TNFα directly modulates TrkB, making it particularly suitable for this experiment (Kim et al., 2023a).

Furthermore, strong evidence suggests that TNF-α upregulates BDNF transcripts, thus contributing to neuroprotection in adverse inflamed scenarios (Bałkowiec-Iskra et al., 2011; Saha et al., 2006). BDNF is a neurotrophin that is involved in various cellular and biological processes such as modulation of the survival of stem cells and progenitors, neurogenesis and neuronal differentiation, the branching and survival of differentiated neurons, and the formation of maturation of the dendritic and synapses (Wu et al., 2016). The transcription of BDNF and expression of its receptor, TrkB, is mediated by the activation of the cAMP response element binding protein (CREB) (Gibon et al., 2013). Previous studies have established that CREB activation is contingent upon the phosphorylation of Ser-133.

Intracellular Ca^2+^ plays a role in initiating the activation of Ca^2+^/calmodulin-dependent protein kinases (CaMK’s), triggering their phosphorylation, thereby facilitating the transcription of BDNF and the expression of TrkB (Yan et al., 2016). The present study focuses on Ca^2+^-calmodulin-dependent protein kinase II (CaMKII), a family of multifunctional Ser/Threonine protein kinases (α, β, γ, and ∂) (Rostas and Skelding, 2023). Activation of this kinase occurs upon the binding of Ca^2+^ to calmodulin. CaMKII is involved in phosphorylating a broad spectrum of target proteins implicated in cellular processes such as cell growth and apoptosis (Salas et al., 2010). While several investigations have suggested a role for CaMKII in bone regeneration through chondrocyte maturation, the exact mechanism(s) remain unclear (Taschner et al., 2008). Previous studies have shown CaMKII to be pro-apoptotic under conditions of enhanced β1-adrenergic stimulation or enhanced Ca^2+^ influx through L-type Ca^2+^ channels, inhibiting CaMKII would alter this effect (Vila-Petroff et al., 2007; Kim et al., 2020). Thus, we hypothesized that hyper-activation of CaMKII may lead to more harm than good. Hence, inhibition of CaMKII could potentially stimulate the differentiation of hDPSCs via TrkB under controlled TNFα-stimulated inflammatory conditions.

## 2 Materials and methods

### 2.1 Chemicals and reagents

Human dental pulp stem cells (hDPSCs) were purchased from Lonza, Pharma & Biotech (Cat #PT-5025). MEM-alpha, DMEM, PBS, fetal bovine serum, L-glutamine, and Antibiotic–Antimycotic were procured from Gibco™ Fisher Scientific (Pasiewicz et al., 2022). Poly-D-Lysine coated (12 mm) round German glass coverslips were purchased from Corning™ Fisher Scientific (Cat #354087). The CaMKII inhibitor (MilliporeSigma, Cat #20-871-1500UG), human recombinant CaMKII protein (Avantor, Cat #103680-002), human TNFα recombinant protein (Invitrogen, Cat #ENRTNFAI), LTA (Fisher Scientific, Cat #50-177-9999), LPS (Invitrogen, Cat #2270741), TrkB receptor agonist; LM22A-4 (R&D System, Cat #4607), and TrkB receptor inhibitor; Cyclotraxin-B (Tocris Bioscience, Cat #50621) were commercially obtained. Various antibodies were purchased: mouse anti-STRO-1 (Santa Cruz, Cat #sc-47733), mouse anti-CaMKII (Sigma, Cat #WH0000815M1), rabbit anti-p-CaMKII (Santa Cruz, Cat #SAB4503756), mouse anti-DMP-1 (R&D System, Cat #K5041-1D4), and rabbit anti-DSPP (Santa Cruz, Cat #SA160926LC). siRNA targeting human CaMKII (Cat #sc-29900-SH), siRNA control (Cat #sc-37007), and siRNA Reagent System (Cat #sc-45064) were purchased from Santa Cruz Biotechnology.

### 2.2 Cell culture and differentiation

Commercially available hDPSCs (Lonza, Cat #PT-5025), which were guaranteed through 10 population doublings to express CD105, CD166, CD29, CD90, and CD73 and do not express CD34, CD45, and CD133, were further evaluated by immunocytochemistry in cultures with the STRO-1, a stem cell marker. The hDPSCs were cultured in normal growth media (10% FBS, 1% L-glutamine, and 1% antimycotic/antibiotic in MEM-α). hDPSCs were kept at 37°C and 5% CO_2_ for 4 days. After normal differentiation, the media were changed to dentinogenic media (DMEM containing 10% FBS, 1% L-glutamine, and 1% antimycotic/antibiotic, 50 μg/mL ascorbic acid, 10 mM β-glycerophosphate, and 10 nM dexamethasone). TNFα (20 ng/mL) was added to induce inflammation on days 4 and 7 for 1 h, right before the change to dentinogenic media. The CaMKII inhibitor (5 μM/mL) or CaMKII protein (1 μM/mL) was treated with dentinogenic media on days 4, 7, 10, and 14. All experiments were conducted with different sets of hDPSCs about 3–4 times and used the second and third passages.

### 2.3 Silencing of CaMKII expression by small interfering RNA (siRNA)

Human DPSCs were grown in 6 well plate culture chambers in 2 mL of free-antibiotic medium up to 70% confluence, and then transient transfection with siRNAs was performed using the siRNA Reagent System according to the manufacturer´s protocol as previously described (Irfan and Chung, 2023). Cells were incubated at 37°C in a CO_2_ incubator in 1 mL of free-antibiotic and free-serum transfection solution containing a mixture of transfection reagent and 40 pmol/mL of CaMKII siRNA or control siRNA, which is a non-targeting siRNA designed as a negative control. After an incubation of 6 h, 1 mL of medium containing 2 times the normal serum and antibiotics concentration was added in each well without removing the transfection mixture. After 24 h, the medium was aspirated and replaced with fresh normal growth medium (DMEM + 4.5 g/L glucose, L-glutamine, sodium, pyruvate + 10% heat-inactivated FBS + 100 μg/mL streptomycin, 100 U/mL penicillin). Assays using siRNA silenced cells were performed within 72 h after adding fresh medium or differentiated for 2 weeks in dentinogenic media.

### 2.4 Quantitative PCR (qPCR)

Human DPSCs were cultured in a 6-well plate at 5 × 10^4^ cells per well. The total mRNA was extracted using RNeasy Mini Kit (Qiagen, Cat #74104), and the containing quantity of cDNA was measured using the NanoDrop 2000 (Fisher Scientific, Cat #ND2000). The Fast SYBR™ Green Master Mix (ThermoFisher, Cat #4385616) was used to identify the cDNA sample according to the manufacturer’s protocol. Primer sequences (Integrated DNA Technologies) were used predesigned hGAPDH (Forward: 5′-GGC ATC CAC TGT GGT CAT GAG-3′, Reverse: 5′-TGC ACC ACC AAC TGC TTA GC-3′), hDSPP (Forward: 5′-CTG TTG GGA AGA GCC AAG ATA AG-3′, Reverse: 5′-CCA AGA TCA TTC CAT GTT GTC CT-3′), and hDMP-1 (Forward: 5′-CAC TCA AGA TTC AGG TGG CAG-3′, Reverse: 5′-TCT GAG ATG CGA GAC TTC CTA AA-3′).

### 2.5 Immunocytochemistry

The seeded hDPSCs were incubated in a 12-well plate with a coverslip at 37°C in a CO_2_ incubator until 70%-80% cell confluency. The coverslips were fixed with 4% paraformaldehyde for 2 h at 4°C, permeabilized and saturated as previously described (Irfan et al., 2022b). For blocking and permeabilization, 10% normal goat serum and 0.01% Triton X in 0.01 M phosphate buffer solution (PBS, 0.1% Triton X in PBS) were used for 1h at RT. For primary antibody treatment, the specimens were treated with mouse anti-CaMKII (1:1000), rabbit anti-p-CaMKII (1:1000), mouse anti-DMP-1 (1:1000), or rabbit anti-DSPP (1:1000), mouse anti-STRO1 (1:1000), diluted in 10% Normal Goat Serum (NGS) PBST for overnight at 4°C. Followed by treatment with Alexa Fluor-594 anti-mouse IgG, Alexa Fluor-488 anti-rabbit IgG (1 μg/mL), and/or DAPI (1 μg/mL) (Fisher Scientific, Cat # PIA32742, A12379, EN62248) for 2 hours. The specimens were mounted on the glass slides, and images were obtained using a Leica microscope.

### 2.6 Enzyme-Linked immunosorbent assay (ELISA)

Supernatants were collected from the differentiated hDPSCs, and a DMP-1 ELISA kit (R&D Systems, Cat #EHDMP1) was used for the experiment according to the manufacturer’s protocol. A standard curve was plotted using the values from the standards at increasing concentrations and samples, and the results were normalized with duplicate test samples from various treatments.

### 2.7 In-cell western assay

Human DPSCs were cultured in 96-well optical-bottom plates and treated with TNFα and other treatments twice a week until day 10 of odontoblastic differentiation. Then, the cells were immediately fixed with 100% cold methanol (15 min) and saturated with 5% BSA (1.5 h). The cells were incubated overnight at 4°C with anti-CaMKII, anti–phospho-CaMKII, or anti-β-actin. The cells were then washed (0.05% Tween-20/PBS) and incubated with the respective IRDye-680RD or IRDye-800RD secondary antibody (1 h). After 5 washes, the plates were scanned at 700 and/or 800 nm (Odyssey CLx).

### 2.8 Alizarin red staining (ARS)

The 14-day differentiated hDPSCs in 12-well plates were washed two times with distilled water and fixed with 4% PFA for 1 hour at room temperature, as previously described (Kim et al., 2023b). The specimens were then washed with distilled water two times, and 1 mL of 40 mM of ARS media (ScienCell, Cat #8678) was added per well for 2 h with gentle shaking. The plate was washed with 1X PBS three times and dried. The cells were inspected using a Leica DMi1 phase microscope, and images were obtained and analyzed by ImageJ software.

### 2.9 Statistical analysis

Statistical analyses were performed using GraphPad Prism (GraphPad Software, RRID: SCR_002798). The statistical analyses were performed on at least 3 or 4 independent experiments with duplicates or triplicates, and statistical significance was determined using one-way analysis of variance (ANOVA) followed by student’s t-test to compare the different treatments and their respective controls (p-value of 0.05 or less was considered statistically significant). In addition, the data were analyzed using Tukey’s test to determine statistical significance between the groups. For the quantification of immunofluorescence staining intensity, ImageJ software 1.5.4V was used (RRID:S CR_003070). Fixed areas of 1 mm × 1 mm or 2 mm × 2 mm were selected to analyze differentiated cells number or fluorescence intensity. Detailed statistics for each experiment are shown in the figure legend.

## 3 Results

### 3.1 Expression of CaMKII and p-CaMKII in hDPSCs is modulated in response to various inflammatory mediators

CaMKII expression and function in DPSCs have not been investigated. Here, we investigated if CaMKII modulation enhances dentin-pulp regeneration after severe carious injury by focusing on the DPSCs odontoblastic differentiation and associated dentinogenesis. To the best of our knowledge, our study will be the first to characterize the role of CaMKII in dentinogenesis mediated by inflammation, which will leverage existing genetic approaches.

**FIGURE 1:**
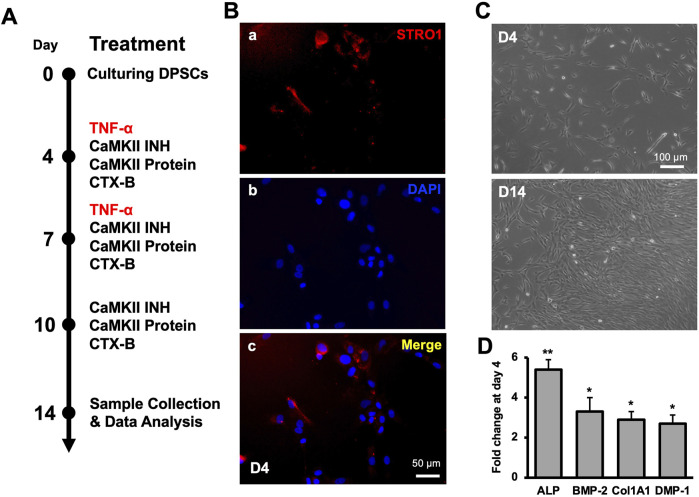
Molecular expression of CaMKII and p-CaMKII in hDPSCs. **(A)** Schematic timeline representation of odontogenic DPSCs differentiation until 14 days of odontoblastic hDPSCs differentiation with various treatments (TNF-α, CAMKII INH, CaMKII P, and CTX-B). **(B)** Expression of mesenchymal-stem-cell marker (STRO-1) and DAPI **(A, B)** with the merged image **(C)** in hDPSCs at day 4. **(C)** Images of early differentiated hDPSCs at day 4 (D4) and after 10 days of differentiation (D14) under the light microscope. Scale bars: **(B)** 50 μm; and **(C)** 100 μm. **(D)** The expression of odontoblastic markers ALP, BMP-2, COL1A1, and DMP-1 mRNA during the odontogenic differentiation was quantified by real-time PCR. The elevated level of these markers represents the odontoblast-like differentiation of DPSCs. The bar graph shows the mean ± SD of at least three independent experiments (n = 3) in duplicates. *p < 0.05 and **p < 0.01 vs fold change at day 0.

First, we examined CaMKII-mediated dentinogenesis during hDPSCs odontogenic differentiation over a 14-day period (Figure 1A, schematic timeline). Commercially available hDPSCs were validated through the mesenchymal stem cell marker, STRO-1 (Figure 1B). An increase in cell numbers was observed on D4 and D14 (Figure 1C). To validate the proposed *in vitro* model for studying odontoblast-like cell differentiation from hDPSCs, alkaline phosphatase (ALP), bone morphogenic proteins 2 (BMP-2), type 1 collagen (Col1A1), and DMP-1 were analyzed using real-time PCR and our results showed significant increment in their expression indicating the odontoblastic differentiation of hDPSCs (Figure 1D; p < 0.05 and p < 0.01).

**FIGURE 2:**
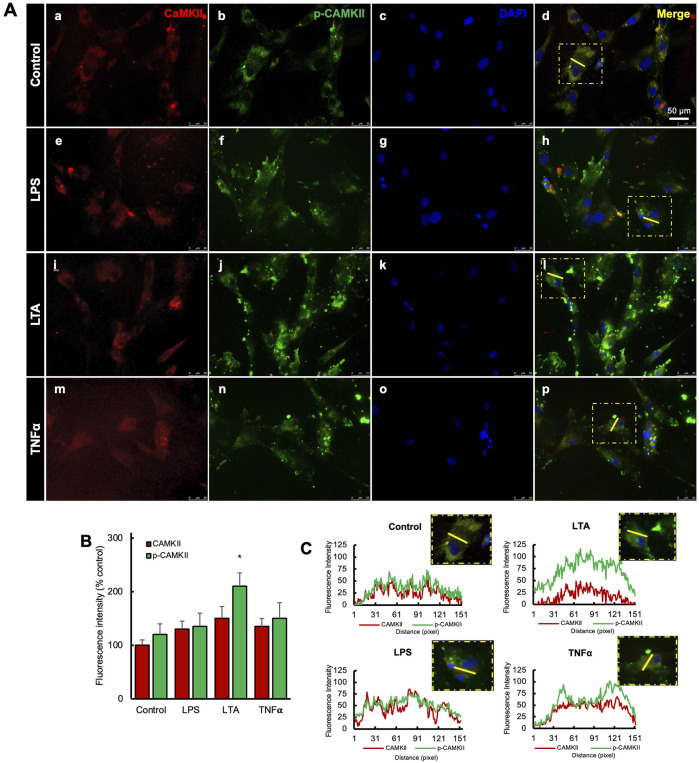
Expression of CaMKII-mediated DSPP and DMP-1 after hDSPCs odontoblastic differentiation. **(A)** Immunofluorescence double staining of DSPP and DMP-1 expression in CaMKII-mediated DPSC differentiation after 14 days. Anti-DSPP (green) expression of control (a), CaMKII inhibitor (f), and CaMKII protein (k). Anti-DMP-1 (red) expression of control (b), CaMKII inhibitor (g), and CaMKII protein (l). Staining nuclei with DAPI (blue; c, h, m). Co-localization of DSPP, DMP-1, and DAPI (d, i, n). Scale bar: 50 μm. Higher magnification of merged images (e, j, o). Scale bar: 50 μm. **(B)** Analyzed anti-DSPP (green) fluorescence intensity from different treatment groups: Control, CaMKII INH, and CaMKII protein. **(C)** Analyzed fluorescence intensity of anti-DMP-1 (red) from the different treatment groups. The bar graph shows the mean ± SD of at least three independent experiments performed in duplicate (n = 3). *p < 0.05, **p < 0.01 vs. control.

Expression levels of CaMKII and p-CaMKII were assessed in hDPSCs when exposed to various inflammatory stimuli (Figure 2). Inflammation was induced using LPS, LTA, and/or TNF-α, resulting in a significant increase in their expression compared to the control (Figures 2Aa–p). A bar graph depicted the upregulation of p-CaMKII in the LTA-treated group, but no significant difference was observed among the other groups. (Figure 2B). Additionally, the integrated intensity of single cells was analyzed in all treatment groups (Figure 2C).

### 3.2 CaMKII inhibitor potentiates the expression of odontogenic markers while CaMKII protein reverses its effect in hDPSCs odontogenic differentiation

**FIGURE 3:**
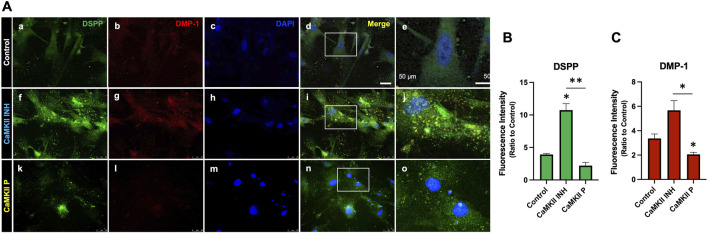
Expression of CaMKII-mediated DSPP and DMP-1 after hDSPCs odontoblastic differentiation. **(A)** Immunofluorescence double staining of DSPP and DMP-1 expression in CaMKII-mediated DPSC differentiation after 14 days. Anti-DSPP (green) expression of control (a), CaMKII inhibitor (f), and CaMKII protein (k). Anti-DMP-1 (red) expression of control (b), CaMKII inhibitor (g), and CaMKII protein (l). Staining nuclei with DAPI (blue; c, h, m). Co-localization of DSPP, DMP-1, and DAPI (d, i, n). Scale bar: 50 μm. Higher magnification of merged images (e, j, o). Scale bar: 50 μm. **(B)** Analyzed anti-DSPP (green) fluorescence intensity from different treatment groups: Control, CaMKII INH, and CaMKII protein. **(C)** Analyzed fluorescence intensity of anti-DMP-1 (red) from the different treatment groups. The bar graph shows the mean ± SD of at least three independent experiments performed in duplicate (n = 3). *p < 0.05, **p < 0.01 vs. control.

To assess the impact of CaMKII on odontogenic differentiation in hDPSCs, we treated cells with either the CaMKII inhibitor (CaMKII INH) or CaMKII proteins (CaMKII P) and analyzed protein and mRNA levels on days 10 and 14 (Figure 3). The treatment groups were distinguished into 3 groups: control, CaMKII INH, and CaMKII P. Anti-DSPP and anti-DMP-1 antibodies were utilized to identify odontoblasts-like differentiation of hDPSCs, indicative of dentinogenesis. Double immunostaining revealed co-expression of DSPP and DMP-1 in hDPSCs (Figure 3A). Results demonstrated a significant increase in DSPP fluorescence intensity levels following treatment with CaMKII INH (6.82 ± 1.30, p < 0.05). In contrast, treatment with CaMKII P (−1.72 ± 0.68) attenuated this effect compared to the control. Similarly, the fluorescent intensity of DMP-1 was potentiated by CaMKII INH (2.666 ± 1.026), whereas treatment with CaMKII P (−0.9482 ± 0.2444, p < 0.05) decreased the intensity (Figures 3B, C).

**FIGURE 4:**
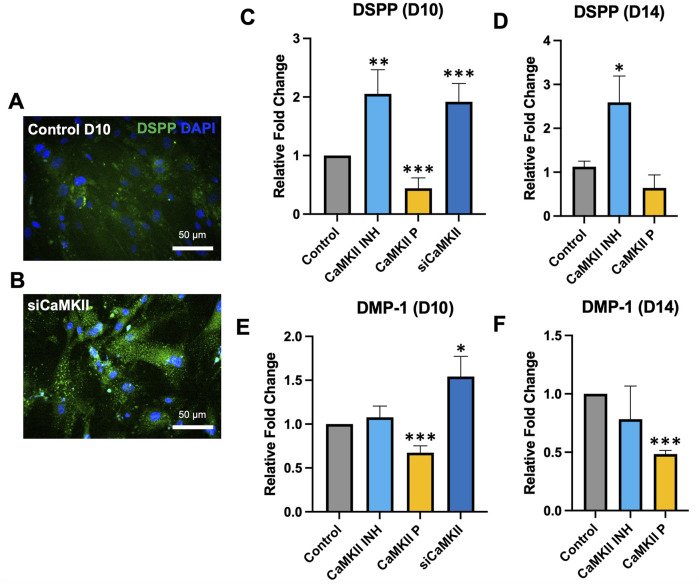
mRNA expression of CaMKII-mediated DSPP and DMP-1 after odontoblastic differentiation and siRNA of CaMKII at days 10 and 14. **(A, B)** Immunopositivity of anti-DSPP expression at day 10 in control and siCaMKII treated odontoblastic differentiated hDPSCs. Scale bar: 50 μm. **(C–F)** mRNA expression of DSPP and DMP-1 in odontoblastic differentiated hDPSCs at day 10 and day 14 from different treatment groups: control, CaMKII INH, CaMKII protein, and siRNA of CaMKII. The bar graph shows the mean ± SD of at least three independent experiments performed in duplicate (n = 3). *p < 0.05, **p < 0.01, and ***p < 0.001 vs control.

To validate whether inhibition of CaMKII potentiates its effects, we utilized small interfering RNA (siRNA) targeting CaMKII to suppress the expression of the CaMKII gene by complementary nucleotide sequence to induce mRNA degradation (Figures 4A, B). mRNA expression of DSPP and DMP-1 at day 10 significantly increased due to CaMKII siRNA (Figures 4C, E), exhibiting a similar trend to the pharmacological administration of CaMKII INH (Figures 4D, F) at day 14. These findings demonstrated the involvement of CaMKII protein and the regulatory impact of the CaMKII gene on both early and late stages of hDPSCs odontogenic differentiation.

### 3.3 CaMKII inhibition enhances hDPSCs odontoblastic differentiation via TrkB under TNFα-induced inflammation

**FIGURE 5:**
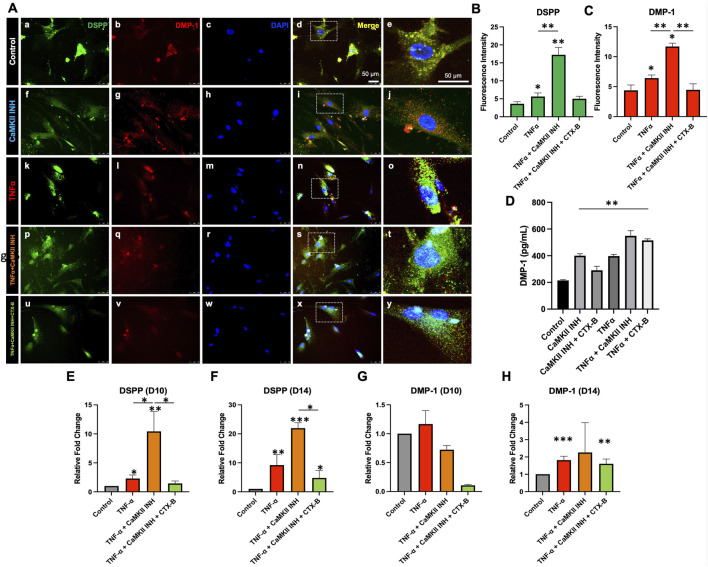
CaMKII-mediated DSPP and DMP-1 expression in TNFα-induced odontogenic hDPSCs differentiation via TrkB receptor. **(A)** Detection of anti-DSPP (green) and anti-DMP-1 (red) in various treatment groups. Anti-DSPP showed in control, CaMKII INH, TNFα, TNFα + CaMKII INH, and TNFα + CaMKII INH + CTX-B (a, f, k, p, u). Anti-DMP-1 is shown in the different treatment groups (b, g, l, q, v). Immuno-positivity of nucleus staining of DAPI (c, h, m, r, w). Merge images of anti-DSPP and DMP-1 (d, i, n, s, x). Scale bars: 50 μm. Higher magnification from the white box of merged images (e, j, o, t, y). Scale bars: 25 μm. **(B, C)** Analyzed fluorescence intensity of DSPP and DMP-1. **(D)** The regulation of the CaMKII affects the odontogenic differentiation of DPSCs. ELISA has compared DMP-1 levels among several treatment groups at day 10 of hDPSCs odontoblastic differentiation. **(E–H)** mRNA expression of CaMKII-mediated DSPP and DMP-1 after TNFα-induced odontogenic hDPSCs differentiation via TrkB at day 10 and day 14. The bar graphs show the mean ± SD of at least three independent experiments performed in duplicate (n = 3). *p < 0.05, **p < 0.01, and ***p < 0.001 vs control.

In our prior investigation, we observed that TNFα-induced inflammation in hDPSCs enhanced odontogenic differentiation mediated by TrkB receptor. We subsequently explored the involvement of the CaMKII molecule in this signaling pathway. The fluorescence intensity of DSPP and DMP-1 significantly increased following exposure to TNFα (2.02 ± 0.66, 2.05 ± 0.61, p < 0.05) and demonstrated further enhancement in the presence of TNFα along with CaMKII inhibition (13.62 ± 1.17, 7.32 ± 0.74, p < 0.05). However, the addition of CTX-B notably diminished this effect (−12.21 ± 1.21, −7.24 ± 0.79, p < 0.05) compared to the TNFα + CaMKII INH group (Figures 5A–C). Further, ELISA results demonstrated higher levels of DMP-1 in TNFα alone and together with the CaMKII inhibitor group (Figure 5D). Our qPCR findings revealed that CaMKII inhibition during TNFα treatment potentiated the expression of odontogenic differentiation markers and the fluorescent intensity of DSPP and DMP-1 (Figures 5E–H). These findings suggest that CaMKII inhibition enhances odontogenic differentiation by activating the TrkB receptor. The TrkB antagonist, CTX-B, can inhibit this effect.

### 3.4 Phosphorylation of CaMKII and mineralization of TNFα-induced odontoblastic differentiated hDPSCs are mediated by TrkB

**FIGURE 6:**
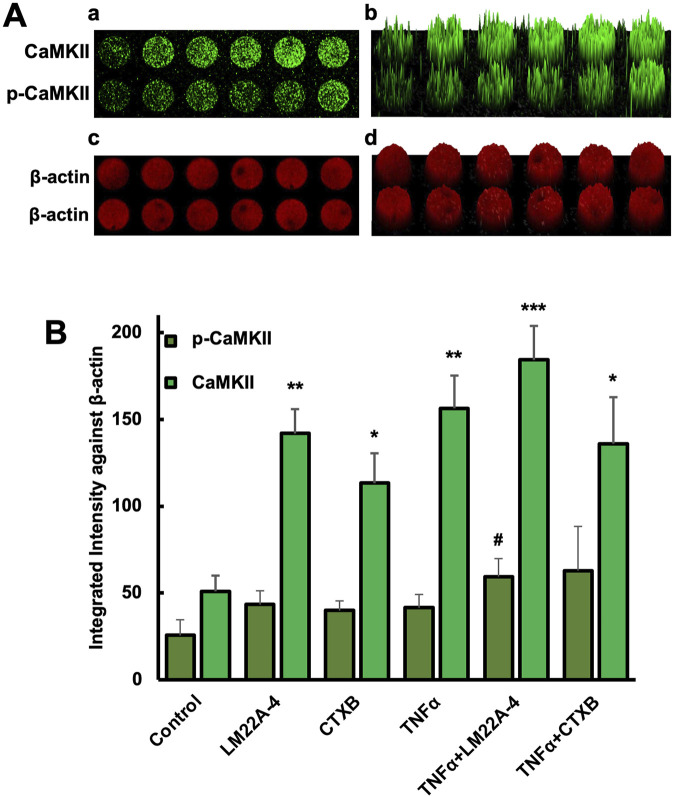
Phosphorylation of CaMKII in DPSC differentiated odontoblast-like cells. **(A)** in-cell western assay showing the expression of CaMKII and p-CaMKII in DPSC differentiated odontoblast-like cells after 10 days of differentiation with various treatments. The expression of CaMKII and p-CaMKII (a) and their corresponding fluorescence intensity distribution in a 3D surface plot (b). The β-actin signal, used as a loading control, showed consistent fluorescence intensity across all samples (Figure A-c), with its 3D surface plot (Figure A-d). **(B)** Bar graph showing the integrated fluorescence intensity. *p < 0.05, **p < 0.01, and ***p < 0.001 vs control. #p < 0.05 vs CaMKII and p-CaMKII.

**FIGURE 7:**
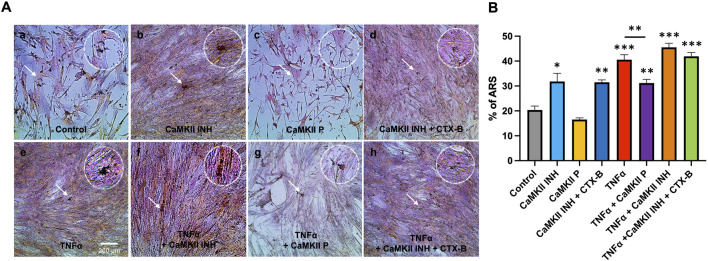
Mineralization activity of CaMKII-mediated odontogenic hDPSCs differentiation by TNFα via TrkB at day 14. **(A)** Representative images of ARS assay from different treatment groups; control, CaMKII INH, CaMKII P, CaMKII INH + CTX-B, TNFα, TNFα + CaMKII INH, and TNFα + CaMKII INH + CTX-B at 14 days of inflammation-induced odontogenic differentiation in hDPSCs. The white arrow indicates calcium crystallization. **(B)** Analysis of the percentages of the stained areas in the ARS assay. The bar graph shows the mean ± SD of at least three independent experiments performed in duplicate (n = 3). *p < 0.05, **p < 0.01, and ***p < 0.001 vs control.

The in-cell western assay showed promising effects of TrkB agonists and antagonists on the expression of CaMKII and p-CaMKII in odontoblastic differentiated hDPSCs potentiated by TNF-α treatment evidencing the interaction of CaMKII and TrkB (Figures 6A, B). The mineralization activity of each treatment group on day 14 (Figure 7) was verified with ARS staining. The white arrows indicate the calcium deposition. Treatment with CaMKII INH led to increased calcium deposition, while CaMKII P treatment resulted in decreased deposition compared to the control, consistent with previous results. Treatment with both CaMKII INH and CTX-B resulted in a slight reduction compared to CaMKII INH alone. Regarding inflammatory stimulation, TNFα amplified its effect compared to the control, and adding CaMKII P diminished this effect. Conversely, the addition of CaMKII INH in TNFα-induced hDPSCs potentiated calcium deposition, while the addition of CTX-B reduced its effect. The calcium deposition, as evidenced by Alizarin Red Staining (Figure 7A white arrows), provides potential evidence of mineralization and dentinogenesis. The inflammatory reaction increased mineralization activity in TNFα-induced hDPSCs differentiation, a process mediated by CaMKII and TrkB. These results indicate that inflammation-induced dentinogenesis is regulated through the CaMKII and TrkB receptor pathways.

## 4 Discussion

The present study elucidates the role of CaMKII inhibition in enhancing hDPSCs odontoblastic differentiation via TrkB under TNF-α-induced inflammation. Our findings expand the understanding of the dentin-pulp complex’s response to controlled inflammation and its potential for regeneration. Human DPSCs are categorized as mesenchymal cells and have been extensively studied and characterized as highly efficient stem cells (Gronthos et al., 2000; Gronthos et al., 2002). When subjected to inflammation, hDPSCs can influence the natural repair process, notably contributing to tertiary dentin formation (Galler et al., 2021). STRO-1 is a general surface marker for mesenchymal stem cells and is expressed in dental pulp stem cells. Its expression is higher in odontoblast-like cells than in hDPSCs (Kang et al., 2019). Therefore, we used hDPSCs to differentiate odontoblast-like cells in our *in vitro* studies. Also, previous studies have proposed the advantages of using hDPSCs over mesenchymal stem cells (Irfan et al., 2022a; Pagella et al., 2020). Our previous studies demonstrated that the BDNF and its receptor TrkB are significant regulators in the inflammation-mediated odontoblastic differentiation of hDPSCs, and its downstream mechanism is related to CaMKII, suggesting a possible linkage between the two in dentinogenesis (Kim et al., 2023a; Irfan et al., 2024; Irfan and Chung, 2023). Our recent finding demonstrated that the activation and inhibition of TrkB alter the odontogenic differentiation of hDPSCs mediated by inflammation, leading to changes in the expression of dentinogenic markers. These modifications in marker expression influence calcium signaling and subsequently affect tertiary dentin formation. Previous studies have demonstrated that CaMKII is one of the downstream effectors of the BDNF/TrkB signaling pathway, and their interaction is involved in mitigating neurotoxicity and memory deficits (Liu et al., 2021; Nagappan and Bai, 2005; Zheng et al., 2020). CaMKII emerges as one of the potentially significant molecules that regulate calcium ions, which are essential for hard tissue formation, including hard tissue regeneration. CaMKII participates in various calcium signaling pathways related to dentinogenesis and osteogenesis (Benchoula et al., 2023; Eapen et al., 2013). Its activation can lead to various physiological abnormalities, such as cell death, vascular hyperpermeability, and impaired vasodilation. Overactive CaMKII function within macrophages has the potential to trigger inflammation, impede efferocytosis, and ultimately culminate in tissue fibrosis (Zhang et al., 2023). An *in vivo* study demonstrated that the absence of CaMKII promotes osteoblast differentiation from mesenchymal stem cells, resulting in increased phosphorylation levels of CREB and PKA in osteoblasts.

Moreover, pharmacological inhibition of CaMKII reduced the formation of multinuclear osteoclasts. Universal genetic elimination of CaMKII has shown beneficial effects on osteoblasts, while detrimental effects are observed in osteoclasts, ultimately leading to an overall increase in bone density (Cary et al., 2013). It is generally accepted that bone and teeth are specialized connective tissues that share similar properties and express profiles of mineralization-related genes (Liu et al., 2024; Ali Abou Neel et al., 2016), and our data presented here are consistent with these previous studies.

We examined the expression of CaMKII and phosphorylated CaMKII (p-CaMKII) following treatment with various inflammatory mediators such as LPS, LTA, and TNFα. Among these inflammatory mediators, TNF-α was particularly emphasized in this study, as this cytokine is known to be induced by LPS and LTA from Gram-negative bacteria, and we can explore a more specific inflammatory pathway (Alan et al., 2022; Koch et al., 2014). This direct pro-cytokine effectively modulates the inflammatory environment. Silencing of CaMKII genes via siRNA involves guiding sequence-dependent slicing of their target mRNAs. By day 10 of siRNA treatment, which represents the longest differentiation period observed under the siRNA effect, siCaMKII exhibited a similar trend of CaMKII inhibition. This confirms that pharmacological administration of CaMKII inhibition was able to yield reliable data without adverse side effects.

**FIGURE 8:**
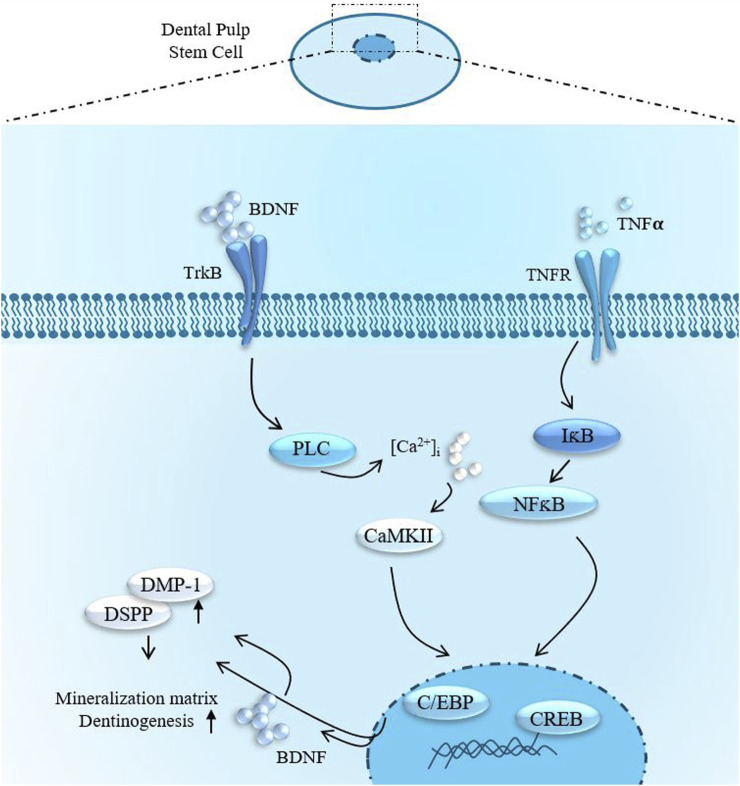
Summarized effects of TNFα-stimulation and BDNF/TrkB signaling in DPSCs odontoblast-like differentiation.

In this research endeavor, we investigated the expression profiles of CaMKII and p-CaMKII expression profiles in hDPSCs. CaMKII and p-CaMKII expression were significantly increased in odontoblast-like differentiated hDPSCs. Junho et al. (2020) (Junho et al., 2020) reported the involvement of CaMKII and inflammation-mediated cardiorenal syndrome. Our findings elucidate that inhibiting CaMKII leads to an enhancement in the expression of odontogenic markers, such as DSPP and DMP-1. Furthermore, we scrutinized the impact of CaMKII protein treatment in hindering this process. Notably, CaMKII inhibition promoted odontogenic differentiation of hDPSCs under both normal physiological conditions and in the presence of inflammatory stimuli. These outcomes highlight the pivotal involvement of CaMKII in various intracellular mechanisms. Additionally, we observed that inhibiting TrkB diminished the effects of CaMKII under TNF-α treatment, suggesting a functional interconnection between CaMKII and TrkB (Figure 8). Cyclotraxin-B (CTX-B) is a selective and high-affinity TrkB antagonist that modulates BDNF signaling, influencing synaptic plasticity, neuroprotection, and neuronal survival. Its ability to cross the blood-brain barrier, along with its anxiolytic, antidepressant-like, and cognitive effects, makes it a promising candidate for studying neurodegenerative diseases and psychiatric disorders. Moreover, CaMKII inhibition increased intracellular calcium levels, which could potentially trigger Ca^2+^-induced BDNF mRNA Transcription under an inflammatory environment.

TNF-α directly activates many dentinogenic pathways (C5aR, TrkB, C5L2, *etc.*). In our recent study, we have studied the role of C5L2 (complement C5a-like receptor; C5aR2) as a negative regulator (Irfan et al., 2022b), which convinced us to look at different aspects of dentinogenesis regulation. Here, TNF-α activates CaMKII; its negative role in dentinogenesis is clear based on our supporting data. However, clinical dentinogenesis occurs in an inflammatory context, so we also investigated CaMK’s modulation under TNF-α. Given that TNF-α may initiate a complex inflammatory response by activating multiple inflammatory and regenerative pathways, including C5aR, TrkB, C5L2, and BDNF (Irfan et al., 2022a; Kim et al., 2023b; Irfan et al., 2024; Irfan and Chung, 2023), further studies are necessary to elucidate their precise roles in caries-induced regeneration.

Despite these findings, our study has certain limitations. The *in vitro* nature of our model may not fully capture the complexity of the *in vivo* microenvironment, including interactions with immune cells and extracellular matrix components. The precise downstream signaling pathways linking CaMKII and TrkB in odontogenic differentiation require further elucidation. Future studies should focus on *in vivo* validation of these mechanisms and explore potential therapeutic applications of CaMKII modulation in regenerative dentistry.

Our studies offer insights into how CaMKII inhibition enhances hDPSCs odontoblastic differentiation via TrkB under TNFα-induced inflammation, shedding light on its underlying mechanism. This study contributes to the growing literature that links calcium signaling, inflammation, and tissue regeneration, offering promising avenues for developing therapeutic strategies for dental caries and pulp tissue repair. These findings hold promise for identifying novel therapeutic pathways for treating dental caries.

## Data Availability

The raw data supporting the conclusions of this article will be made available by the authors, without undue reservation.
